# Assessing institutional capacities to demand and use nutrition data for decision-making in Nigeria’s health sector: A mixed-methods study

**DOI:** 10.1186/s12961-025-01387-9

**Published:** 2025-09-29

**Authors:** Elyse Iruhiriye, Olutayo Adeyemi, Yetunde Akinmolayan, Padmini Vishwanath, Daniela Rodriguez, Rebecca Heidkamp

**Affiliations:** 1 Department of International Health, Johns Hopkins Bloomberg School of Public Health, 615 N. Wolfe Street, Baltimore, MD 21205 USA; 2 University of Ibadan, Ibadan, Nigeria; 3 Nutrition, Agriculture and Health Initiative, Abuja, Nigeria; 4 International Food Policy Research Institute, Washington, DC USA; 5 Ashoka University, New-Delhi, India

**Keywords:** Africa, Data demand, Data use, Enabling environment, Health systems strengthening, Nutrition, Policy process

## Abstract

**Background:**

Using data for policy design, program implementation and accountability is a priority among nutrition stakeholders in Nigeria. However, the capacities of decision-makers to use data are not well-defined.

**Objective:**

This study used mixed methods to assess the capacity of institutions within Nigeria’s health sector to demand and use data for decision-making on nutrition policies and programs.

**Methods:**

A quantitative scale capturing organizational and individual factors related to the capacity to demand and use data was administered to 92 nutrition stakeholders in Nigeria across federal government (*n* = 33), state government (*n* = 21) and local government areas (LGAs) (*n* = 29) and development partner organizations (*n* = 9). We compared scores across sub-groups. Key informant interviews (KIIs) with a subset of the federal (*n* = 13), state (*n* = 17), LGA (*n* = 30), and development partner (*n* = 11) respondents complemented the quantitative scale and were analysed thematically.

**Results:**

Mean institutional capacity to demand and use data was 78.6 out of 100 [95% confidence interval (CI) 75.9, 81.3]. The mean organizational capacity score was 51.4 out of 60 (95% CI 49.9, 52.9); individual capacity was 27.2 out of 40 (95% CI 25.7, 28.7). Development partners (mean 85.7; 95% CI 78.9, 92.4) had the highest score, followed by state-level respondents (mean 82.3; 95% CI 76.9, 87.6), but differences were not significant. Both quantitative and qualitative results showed recognition and support for nutrition data demand and use but weak organizational mechanisms to ensure data use. Accessing available nutrition data was a challenge, especially for administrative data. Quantitative and qualitative results identified infrastructural and technological resource barriers for government respondents, especially at the LGA level, but not for development partners. Skills to synthesize and use nutrition data were also a challenge across respondent groups.

**Conclusions:**

Government and non-government stakeholders in Nigeria’s health sector recognize the importance of data for nutrition decision-making, but gaps remain in individual capacity, resources and data use processes. To strengthen data use for nutrition policy process, investments to address gaps are needed.

**Supplementary Information:**

The online version contains supplementary material available at 10.1186/s12961-025-01387-9.

## Introduction

Addressing malnutrition in all its forms remains a global priority but progress towards global targets including the World Health Assembly Nutrition Targets and Sustainable Development Goal (SDG) 2 has been slow [1]. In 2014 the Global Nutrition Report called for a “nutrition data revolution” to strengthen the availability and use of data to monitor progress, guide investments and drive more effective actions across countries [2, 3]. Since this call to action, there has been notable progress in filling gaps in data collection for diet quality, micronutrient status and health sector nutrition intervention coverage [4–6]. However, understanding of the use of data in the nutrition policy process remains limited in many low and middle-income country (LMIC) contexts [7].

Data demand and data use refer to “an approach in which data and information are collected in response to an identified need, a need justified in terms of the decision-oriented use to which the information will be put.” [8, p. 16]. The capacity to identify data needs, collect and seek relevant information and assess the data to inform program and policy decisions is important to the overall process of data-informed decision-making [9]. This capacity goes beyond individual skills and reflects the interaction between individual, organizational and systems factors [10].

Data are one of many factors, including values, resources, governance traditions, organizational environment and technical capacity, that inform decisions related to policy and programming [11–13]. Decision-makers’ beliefs about and perceptions of data or evidence influence their use [14]. Data need to be timely, be seen as relevant, be of high quality and be accurate [15]. Studies have found that failure to package and present data in an understandable and compelling format and the lack of participation by decision-makers in health information systems design are barriers to data use [16, 17].

The literature on the demand for and use of data in global health has largely focused on the broader health information system or specific domains such as family planning, human immunodeficiency virus (HIV), immunization and maternal and neonatal health [18–23]. There has also been more focus on the use of research evidence to inform nutrition policy than on the use of data from national monitoring and information systems [12, 16, 24]. Over the last 15 years, the health systems field shifted from a data dissemination paradigm, which focused on making data and evidence available, to a data utilization model, which prioritizes translating data and evidence on the basis of the needs of decision-makers to facilitate their use [2, 8, 17, 25].

**Fig. 1:**
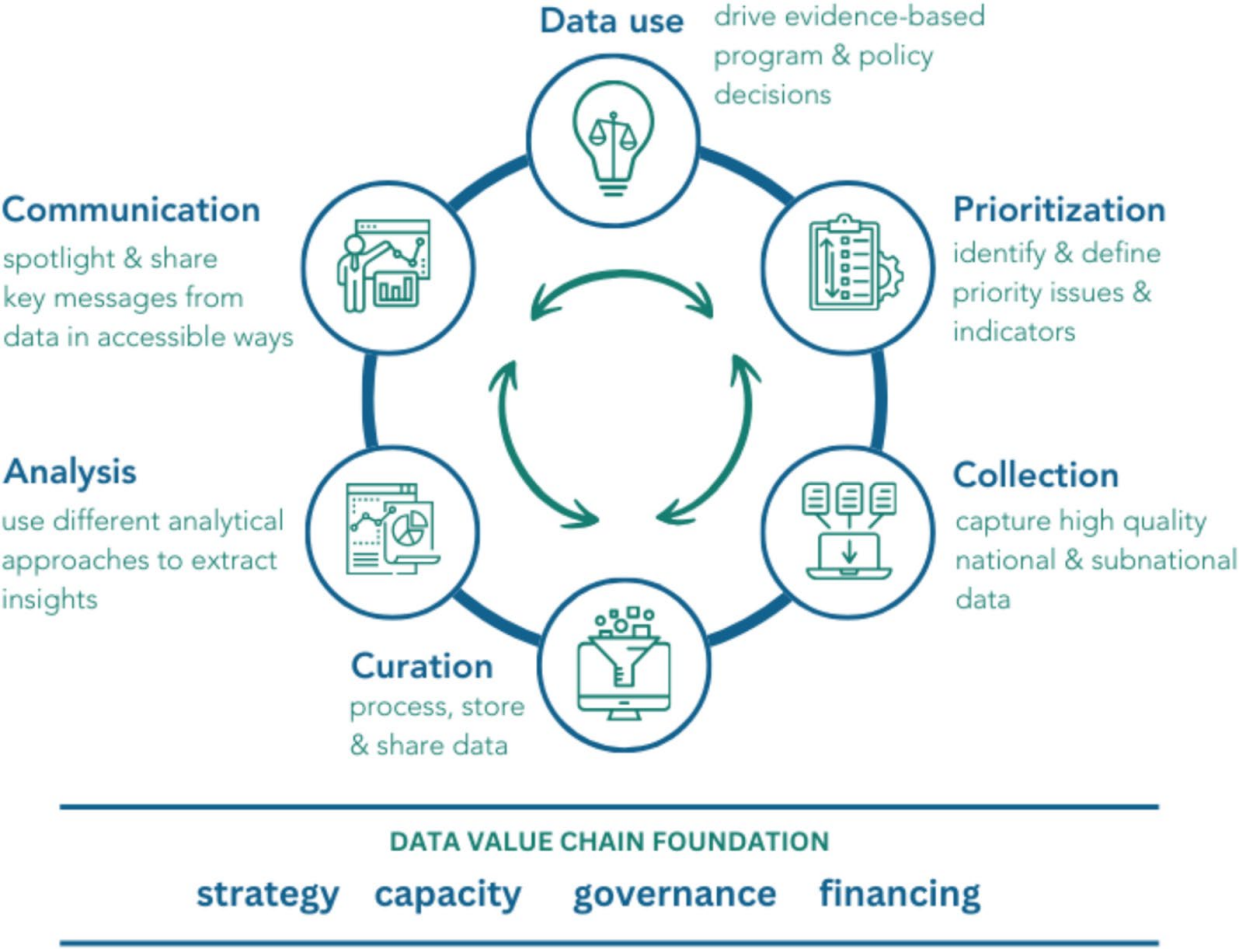
Value chain model for nutrition data

Data demand and use specifically by the nutrition sector has been more explored in high-income countries than low-income countries [14, 26–28]. The few studies in LMICs have focused on national-level decision-makers, with limited attention to subnational levels, where important decisions around strategic planning, program and policy implementation and monitoring and evaluation also occur [12, 16, 24, 29]. One available study from India that included subnational-level decision-makers, found that less than a quarter (23%) of respondents used data to inform decisions due to challenges related to human resources, technology infrastructure and political structures and low capacity [30]. Other nutrition literature has identified gaps in nutrition-relevant data from non-health sectors, poor quality of administrative data and an unmet need for capacity strengthening around data use as barriers [6, 16, 31]. The nutrition sector has recently adopted a circular data value chain that positions data use as both an outcome and a stimulus for other links of the value chain, such as data prioritization and data collection (Fig. 1). Similar to the health systems data utilization model, the nutrition data value chain emphasizes the importance of data translation by synthesizing and communicating actionable information for decision-makers (for example, recommendations).

Our study aims to further our understanding of data use in the nutrition policy process in LMICs by (1) assessing the capacity of national and subnational institutions in Nigeria’s health sector to demand and use nutrition data to inform decision-making and (2) to identify the factors that facilitate and constrain this capacity. Nigeria is a country with persistently high burdens of malnutrition [32–34]. Since 2017, the nutrition sector in Nigeria has prioritized investment in data, including filling data gaps around food consumption and micronutrient status and developing a multi-sector nutrition information system (NIS) [35]. Several studies assessing availability, quality and use of data in Nigeria’s health sector found challenges with the completeness and timeliness of data for malaria, immunization and maternal, newborn and child health programs [24, 36–38]. No studies specific to nutrition are available. Our study was conducted as part of a larger effort to develop recommendations for the health sector about nutrition data collected in Nigeria [35, 36].

## Methods

### Study design and setting

This study used mixed methods to assess data demand and data use among national and subnational government and development partner institutions working on nutrition in the health sector in Nigeria. The study was conducted at the federal level and in two states: Kaduna in the north and Lagos in the south. The two purposively selected states fit four pre-defined criteria: geographic diversity (that is, north/south), high levels of nutrition investment, perceived high levels of political commitment for strengthening data systems and low security risk for in-person data collection. Nigeria’s northern and southern states have deep-rooted differences in their social, cultural, economic and political contexts as well as in health system performance and utilization [37, 38]. Given the potential influence of these factors on policy decision-making our study included both a northern and southern state to allow for nationally relevant recommendations. In each state, the study team consulted with the State Nutrition Officer (SNO) to select two local government areas (LGA). The SNOs selected the LGAs that, based on their personal knowledge, had timely reporting of data to administrative systems [for example, District Health Information System (DHIS-2)] and functional cadres delivering community-level nutrition interventions.

### Survey scale

**Fig. 2:**
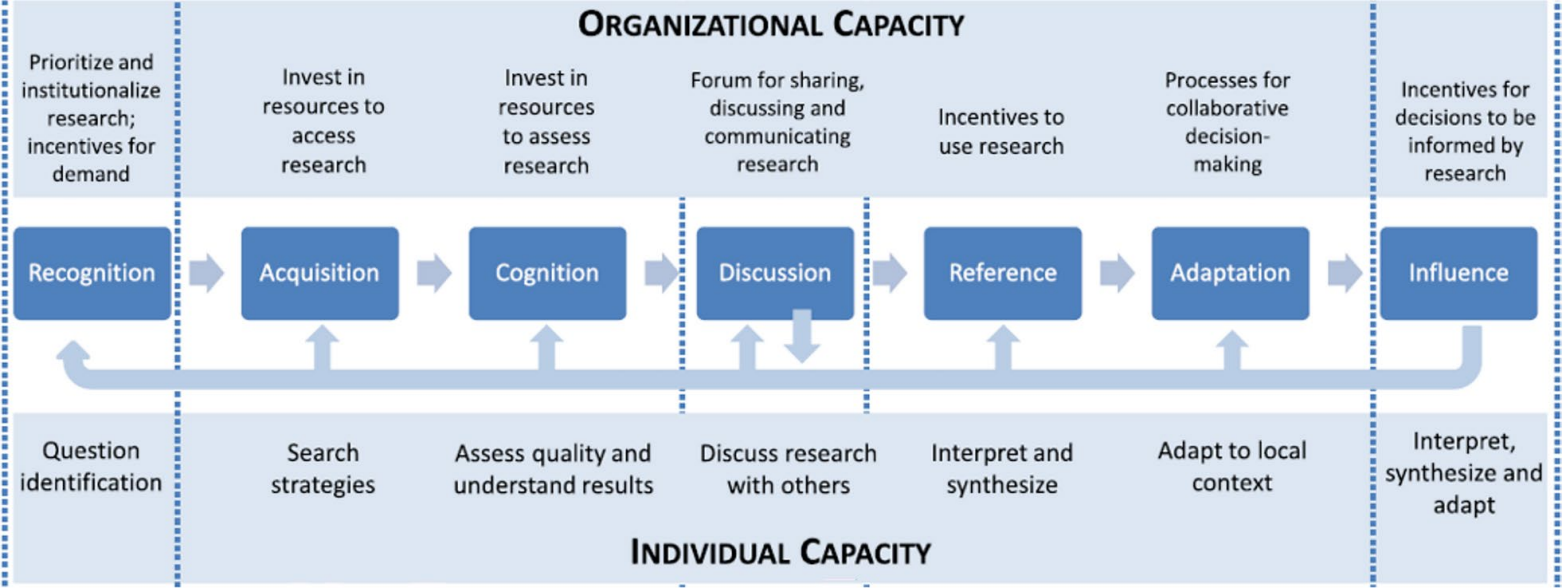
Conceptual framework for the capacity to demand and use evidence (This figure is adapted from Rodriguez et al. [10] originally published in *Health Research Policy and Systems* by Springer Nature)

To assess the capacity to demand and use data, we adapted a framework and survey scale originally developed by Rodríguez and colleagues to measure capacity to demand and use research evidence for decision-making among ministry of health officials in LMICs [10]. Informed by Landry et al. [39], the framework depicts seven steps along the research demand and use process, distinguishing between steps related to demand and those related to use (Fig. 2). The first two steps, recognition and acquisition, represent demand. The subsequent steps of cognition, discussion, reference, adaptation and influence reflect use (Table 1). Although the steps are presented sequentially, the process itself is neither linear nor fixed because decision-making is inherently non-linear and iterative. The framework (Fig. 2) depicts this dynamic nature through feedback loops that indicate possible pathways between steps.

**Table 1:** Seven steps needed in the process of demanding and using data. Table adapted from Landry et al. [39] and Rodriguez et al. [10]

	Steps	Definition
Demand	Recognition	Motivation to use data; can identify questions that can be answered by data. Use of data is prioritized and institutionalized.
	Acquisition	Knowledge of where and how to search for data. Resources available to access data.
Use	Cognition	Ability to assess quality and understand data. Resources available to assess data.
	Discussion	Data are discussed with others such as colleagues and researchers. There are fora to share, discuss and communicate data.
	Reference	Ability to interpret and synthesize data. Incentives exist to use data.
	Adaptation	Ability to adapt results to local context and there is a process for collaborative decision-making.
	Influence	Sufficient latitude within their role to use data to influence decisions. And incentives exist for decisions to be informed by data.

At each step, the framework identifies relevant capacities at individual (that is, skills to identify and interpret research), organizational (that is, structures and practices that support demand and use) and systems levels (that is, leadership to influence broader policy environment) [10, 39]. The survey scale reflects two of these levels: organizational and individual. The scale was validated with ministry of health staff in eight LMICs, including South Africa, Zambia and Bangladesh, and has been used subnationally in India (10; personal communication with Dr. Daniela Rodríguez).

The survey tool for the scale includes 30 Likert scale items [10]. The organizational factor consists of 20 items to assess processes and practices that reflect institutional commitment to demand and use data. The individual factor is made up of 10 items and evaluates individual skills to use data and to provide data-informed recommendations.

Our study team made two major adaptations to the scale developed by Rodríguez et al. The items in the scale were changed to reflect “data use” rather than “research evidence use”, given our more specific focus on use of data from national monitoring and information systems in Nigeria. Research evidence is a broader concept that can entail evidence from data, evaluation studies or implementations science studies [11, 40]. Second, the original scale was designed for use with government institutions, but we also administered the scale to development partner respondents [for example, nongovernmental organizations (NGOs)], which play an important role in addressing nutrition in Nigeria [41].

### Key informant interviews

Qualitative semi-structured key informant interviews (KIIs) were conducted with a sub-set of respondents at the federal, state and LGA levels. The KIIs provided in-depth information about data demand and use to complement the scale and address other elements of the data value chain that influence data-informed decision-making. Interview guides covered topics including the types of decisions respondents make regularly and the use of data in those decisions, the perception of data availability and access, data gaps and the broader enabling factors that influence data demand and data use. The guides developed for LGA monitoring and evaluation (M&E) officers and health facility staff covered a more focused set of topics, including data generation, data quality, data use and feedback on collected data given their role on the front line.

### Sampling and recruitment

We used a purposive approach to identify study respondents for both the survey and KIIs. Respondents were individuals from government institutions in or adjacent to the health sector and development partner institutions that fund or implement nutrition-related programs. Government respondents represented different types of institutions, including the Federal Ministry of Health (FMOH) and State Ministry of Health (SMOH), whose mandates include policy development, supervision and monitoring and evaluation, and the National Primary Health Care Development Agency (NPHCDA) and State Primary Health Care Development Agency (SPHCDA), which are semi-autonomous institutions focused on service provision, program implementation and capacity strengthening [35, 42]. LGA-level respondents included Supervisory Councilors for Health, Department of Health Officers, LGA M&E officers and various facility-level actors including nutrition focal points and health facility staff. Three government respondents at the federal level and one at the state level had roles related to nutrition but were not explicitly in the health sector. Other respondent-level inclusion criterion were that their professional role included making decisions related to nutrition policy or program and they had worked in Nigeria at least 3 years.

The quantitative survey scale was administered to 92 total participants from federal (*n* = 33), state (*n* = 21), LGA (*n* = 29) and development partner (*n* = 9) institutions. The potential number of respondents was limited by the number of stakeholders in the health sector at each level that were involved in nutrition activities and decision-making. A total of 71 KIIs were then conducted with federal (*n* = 13), state (*n* = 17), LGA (*n* = 30) and development partner (*n* = 11) respondents. In some cases, multiple people from the same institution participated in a single interview. Respondents completed the survey scale first to avoid biasing their responses. The KII was conducted with the different stakeholders until data saturation was reached at each level. Data saturation is the point at which additional interviews do not yield any new information, and it is recommended that additional interviews are not conducted after this [43].

There were five respondents who participated in a KII without completing the survey scale because of their time constraints. These five respondents comprised two federal government, one LGA and two development partner respondents. Of all the respondents, 21 completed only the survey scale because data saturation had been reached. Eight of the invited respondents (four federal and four state) did not respond to multiple interview requests.

All government respondents were recruited through formal letters sent to the heads of relevant ministries, departments and agencies (MDAs) involved in nutrition as identified by the study co-author (O.A.), who is based in Lagos and has worked extensively in the nutrition sector. Development partners were contacted directly through email. The letters and emails presented the aims of the study, introduced the research team and requested interviews with relevant actors. Once high-level approval was received, the data collection team followed up with a focal point in each MDA to identify respondents. Development partners identified and selected the relevant respondents for their organizations.

### Data collection

The survey scale instrument and KII interview guides were pilot tested at the federal, state and LGA levels; revisions were made accordingly. On the basis of the pilot testing, the survey scale was administered in English to all respondents, given that all types of respondents comprehended the questions well. Interviewers read the scale items aloud and marked on paper forms the respondents’ level of agreement using a four-point scale ranging from low agreement to high agreement. Responses were entered into Excel after data collection. KII guides were translated into Hausa for Kaduna state and Yoruba for Lagos State, the respective local languages for these states. Respondents at the LGA level were more comfortable participating in an interview in their local language compared with English. Translated interview guides were back-translated into English by a different translator to ensure the instruments retained their intended meaning.

Data collectors were recruited by the University of Ibadan locally in each state to allow in-person data collection and minimize language barriers. They were trained on qualitative methods, the interview and survey tools, research ethics and coronavirus disease 2019 (COVID-19) safety protocols. Data collectors conducted both the survey scale and KIIs.

Data collection was conducted between March and September 2021. Most federal and state survey assessments and KIIs were conducted remotely (via Zoom or WhatsApp) by the lead author (E.I.). Several federal-level surveys were conducted face-to-face by a locally recruited consultant. All LGA- and facility-level interviews were conducted in-person by the trained data collectors. All participants received a consent letter that described study aims, respondents’ rights and the research team. Prior to the start of the interview, respondents provided signed consent to participate in the study and to audio record their interview.

### Analysis

The survey scale was scored using the same approach as the original tool [10]. The 20 organizational factor items were scored from 0 (lowest) to 3 (highest) and were summed to produce a maximum organizational factor score of 60. The ten individual factor items were scored 1 (lowest) to 4 (highest), for a maximum individual factor score of 40. The individual and organizational scores were summed for a maximum overall score of 100. We also analysed individual scale items based on binary outcomes, combining responses for “strongly agree & agree” and “disagree & strongly disagree”, and then assessed the level of agreement on different items. We calculated the mean and 95% confidence interval (CI) for all respondents and by sub-groups including gender, time working in sector (< 20 years versus ≥ 20 years) and institution type (that is, policy development mandates [for example, FMOH/SMOH) versus service provision (for example, NPCHDA/SPCHDA)]. For cross-group comparisons, we interpreted statistical significance on the basis of non-overlapping 95% CIs. All quantitative analyses were conducted using STATA 17.

The KIIs were transcribed and concurrently translated to English as necessary. To analyse the data, an a priori coding list was developed on the basis of the study instruments. E.I. and O.A. used this a priori list to code four transcripts with allowance for emergent codes; all codes were then reviewed and finalized. E.I. and Y.A. analysed KIIs using thematic analysis, which consists of examining data to identify common themes, and met regularly to review analysis and agree on themes. Qualitative data analysis was conducted using NVivo 13. To facilitate interpretation, identified themes were mapped to the seven steps of the data use conceptual framework underlying the survey tool. Data from across the administrative levels were reviewed together, and similarities and differences across levels were noted. We held two separate in-person validation meetings with KII respondents from federal and state/LGA levels to review initial findings and inform interpretation of results.

### Ethics approval

This study received ethics approval from the National Health Research Ethics Committee of Nigeria (NHREC/01/01/2007-08/02/2021) and the institutional review board of Johns Hopkins Bloomberg School of Public Health.

## Results

### Survey scale

About half of the 92 survey scale respondents were female (48%) (Table 2). The proportion of female respondents was lower at the federal level (36%) compared with the LGA (59%) and state levels (52%). Most of the respondents were highly experienced with an average of 18 years working in their sector (range 2–39 years) and 4 years in their current position (range 1–12 years).

**Table 2:** Respondent characteristics

Respondents	*N* (%)	Percent female (%)	Mean time (range) in sector (years)	Mean time (range) in current position (years)
Federal	33 (40%)	36.4	16.6 (2, 39)	4.7 (1, 12)
State	21 (25%)	52.4	22.0 (6, 36)	3.6 (1, 11)
LGA	29 (35%)	58.6	18.3 (2, 35)	3.5 (1, 12)
Development partner	9 (10%)	44.4	14.9 (7, 25)	3.0 (1, 6)
Total	92 (100%)	48.0	18.1 (2, 39)	3.9 (1, 12)

**Fig. 3:**
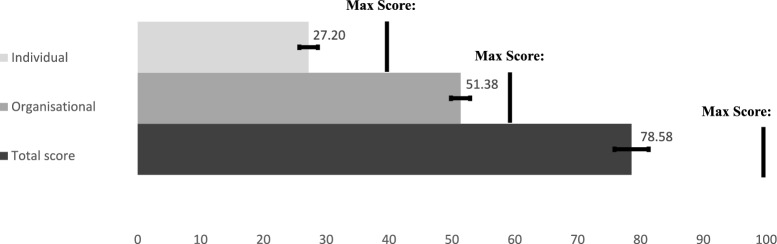
Mean institutional capacity score and factor scores for all respondents

The institutional score is the sum of the organizational score and the individual score. We present mean scores in Fig. 3 and Tables 3 and 4 and then detail item-specific responses for the organizational score and individual score in Tables 5, 6, 7, 8.

**Table 3:** Institutional capacity to demand and use data scores across administrative levels and in development partner institutions

Respondents	Organizational score Mean (95% CI)	Individual score Mean (95% CI)	Total score (institutional score) Mean (95% CI)
Federal (*n* = 33)	49.8 (47.5, 52.1)	27.4 (25.2, 29.7)	77.2 (73.0, 81.4)
State (*n* = 21)	53.6 (50.6, 56.6)	28.7 (25.7, 31.7)	82.3 (76.9, 87.6)
LGA (*n* = 29)	52.1 (49.2, 54.9)	25.8 (23.1, 28.6)	77.9 (72.9, 82.9)
Development partner (*n* = 9)	52.0 (47.6, 56.4)	33.7 (30.6, 36.8)	85.7 (78.9, 92.4)
Total (*n* = 92)	51.4 (49.9, 52.9)	27.2 (25.7, 28.7)	78.6 (75.9, 81.3)

**Table 4:** Institutional capacity to demand and use data scores by gender, experience and health institutions

Respondents	Organizational score Mean (95% CI)	Individual score Mean (95% CI)	Total score (institutional score) Mean (95% CI)
Gender			
Male (*n* = 48)	50.8 (48.6, 53.0)	27.8 (26.0, 29.6)	78.6 (75.1, 82.2)
Female (*n* = 44)	52.0 (49.8, 54.2)	26.5 (24.1, 28.9)	78.5 (74.3, 82.8)
Experience			
< 20 years (*n* = 55)	49.6 (47.5, 51.7)	26.0 (24.0, 28.0)	75.6 (72.0, 79.3)
≥ 20 years (*n* = 37)	54.0 (52.0, 56.0)	29.0 (26.8, 31.2)	83.0 (79.2, 86.7)
Health institution			
Ministry (*n* = 25)	48.4 (45.3, 51.4)	24.4 (21.6, 27.2)	72.8 (67.4, 78.2)
Primary healthcare (*n* = 46)	53.0 (50.9, 55.1)	27.2 (25.2, 29.2)	80.2 (76.5, 83.9)

**Table 5:**
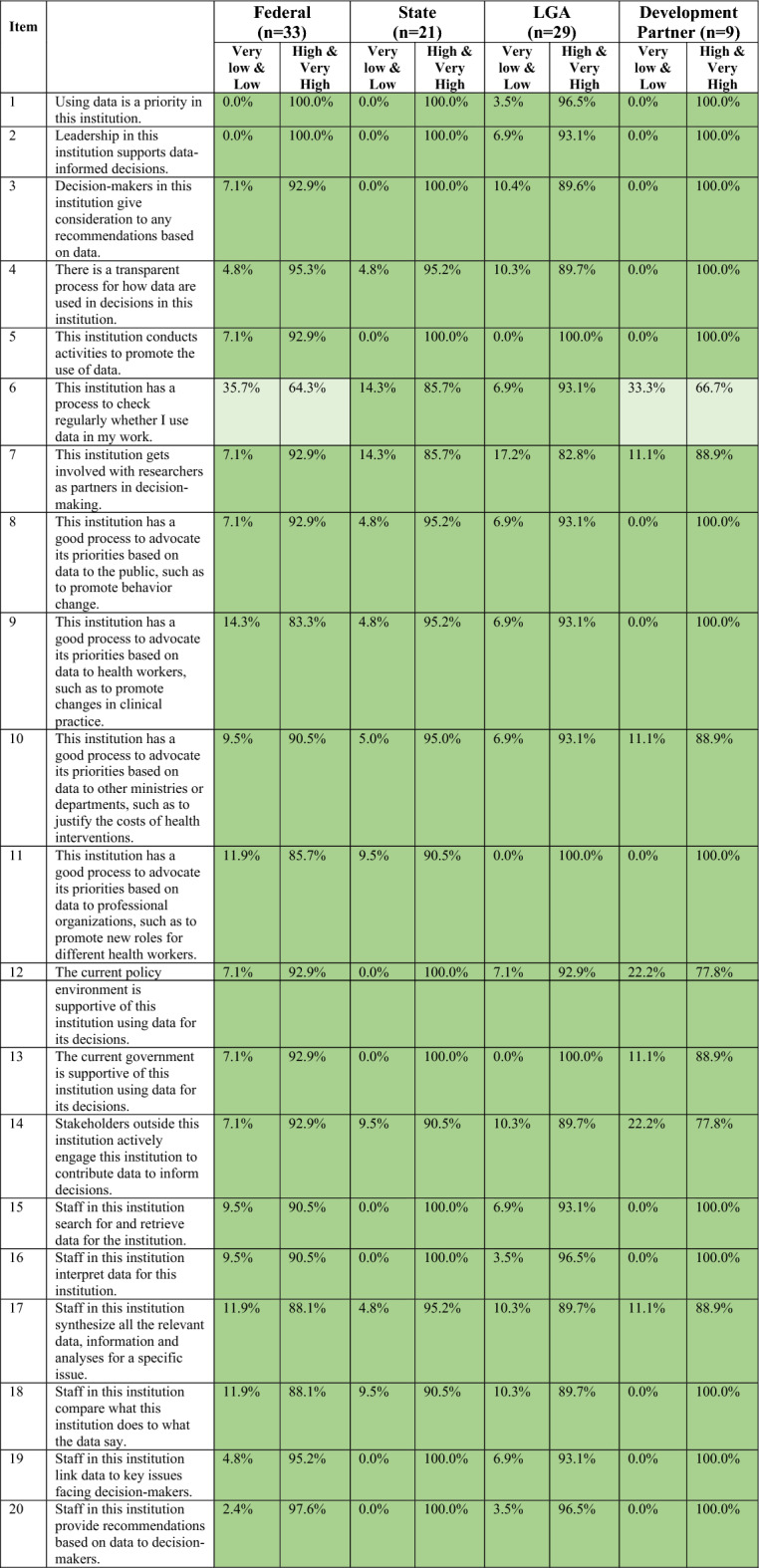
Distribution of scores on organizational capacity items by administrative level and development partners

Key: Levels of agreement

**Table 6:**
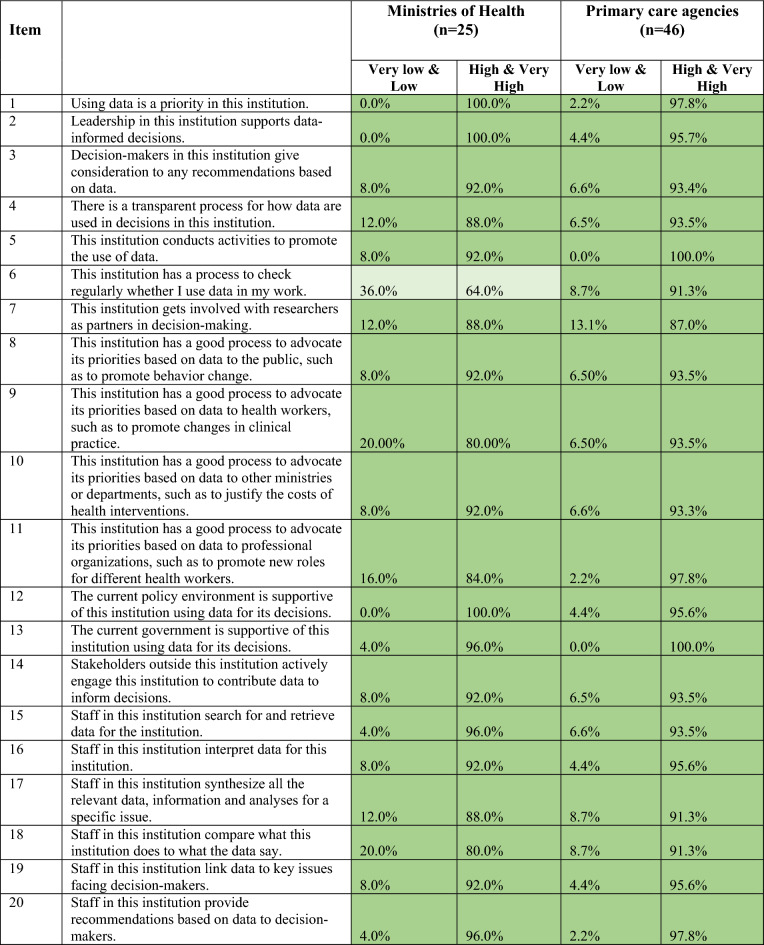
Distribution of scores on organizational capacity factor by ministry and primary care institutions

Key: Levels of agreement

**Table 7:**
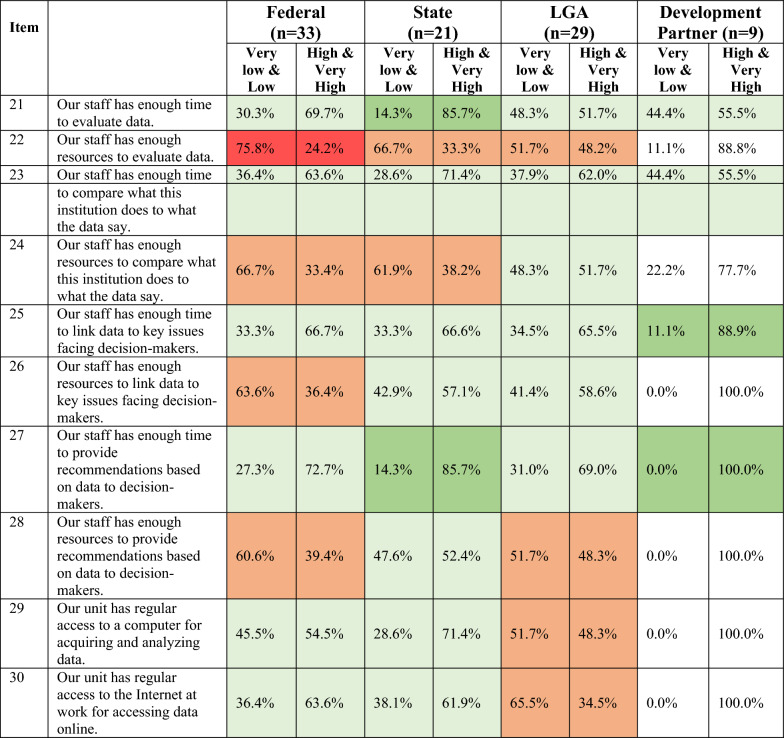
Distribution of scores on individual capacity factor by administrative level and development partners

Key: Levels of agreement

**Table 8:**
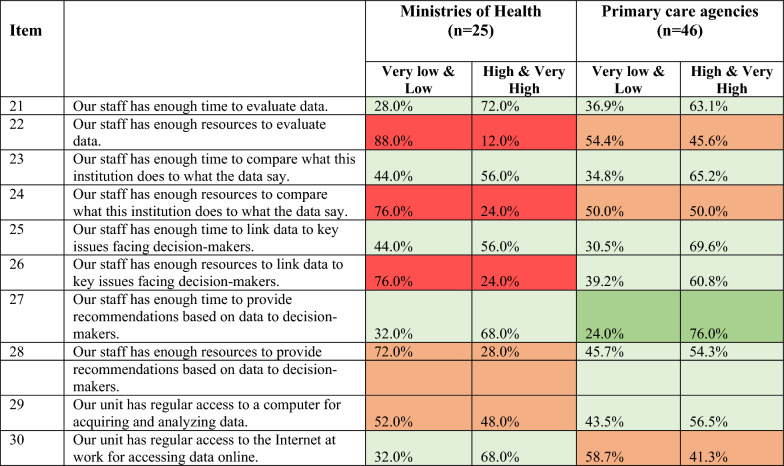
Distribution of scores on individual capacity factor by ministry and primary care institutions

Key: Levels of agreement

For capacity to demand and use data, the mean institutional capacity score was 78.6 out of 100 (95% CI 75.9, 81.3) (Fig. 3). Development partner institutions (mean 85.7; 95% CI 78.9, 92.4) scored themselves highest on institutional capacity to demand and use data, followed by state-level institutions (mean 82.3; 95% CI 76.9, 87.6) (Table 3). Differences in institutional capacity scores were not significant across all sub-groups.

Scores varied by respondent experience and government institution type, although differences were not statistically significant (Table 4). Stakeholders with 20 or more years of experience scored their institutions higher in institutional capacity to demand and use data (mean 83.0; 95% CI 79.2, 86.7) compared with those with less than 20 years of experience (mean 75.6; 95% CI 72.0, 79.3). For the core health sector institutions, implementation agencies (that is, primary healthcare) (mean 80.2; 95% CI 76.5, 83.9) reported higher institutional capacities to demand and use data compared with policy-focused government institutions (that is, ministries) (mean 72.8; 95% CI 67.4, 78.2).

### Organizational capacity items

The mean organizational score was 51.4 out of 60 (95% CI 49.9, 52.9). State-level institutions (mean 53.6; 95% CI 50.6,56.6) scored themselves highest on organizational capacity to demand and use data (Table 3). Federal-level institutions scored the lowest (mean 49.8; 95 CI 47.5; 52.1). The organizational capacity score was not significantly different across groups.

Respondents generally had high levels of agreement on organizational capacity items (Tables 5, 6). Most of the 20 items were scored with high or very high levels of agreement. Nearly all respondents highly agreed that data were a priority in their institutions (item #1) and that leadership supports data use to inform decision-making (item #2) (Tables 5, 6). Respondents from federal institutions were less likely than other groups to highly or very highly agree that their institutions conducted activities to promote the use of data (Item #5); 7% of federal-level respondents scored the item as low (Table 5; Electronic Supplementary Material (ESM) Supplemental 1. Over 90% of respondents from government institutions across administrative levels highly or very highly agreed that the government (item #12) and policy environment (item # 13) are supportive of data use in decision-making. In contrast, 20% of development partners scored items around support for data use (item #12 and items #13) as low or very low.

Respondents had lower levels of agreement on items related to processes for data use within their institutions, including items on verifying whether data are used (item# 6) and how data align with institutional work (item# 18) (Tables 5, 6). A third of respondents from federal institutions and development partner institution respondents scored whether their institutions regularly checked whether data were used in their work (item# 6) as very low/low. In regard to the core health sector institutions, respondents from the policy-focused agencies (that is, ministries) were much more likely to score this item (#6) very low/low compared with respondents from implementation-focused agencies (that is, primary care) (Table 6). Across all types of institutions, about 10% of respondents scored whether institutions had staff to synthesize data (item# 17) as very low/low. Items on collaboration and cross-group discussions about data generally scored very low/low. About 15% of respondents from state and LGA institutions scored working with researchers as partners in decision-making (item #7) as very low/low. One in ten government respondents and 20% of development partners scored whether their institutions shared data with outside stakeholders (item #14) as very low/low.

Across institution types, respondents from policy-focused government institutions ranked items on institutions having good data-informed advocacy processes for health workers (item #9) or professional organizations (item #11) very low/low (~20%).

### Individual capacity items

The mean individual score was 27.2 out of 40 (95% CI 25.7, 28.7). Levels of agreement across the different types of institutions varied more for the individual factor items compared with the organizational factor items (Tables 7, 8; ESM Supplemental 2). Development partner institutions (mean 33.7; 95% CI 30.6, 36.8) had the highest individual capacity score, but this was not significantly higher than other sub-groups. For government institutions, individual capacity scores were slightly higher for state institutions compared with those at the federal and LGA levels, but differences were also not significant.

The item with the highest level of agreement across institutions was whether staff have enough time to provide recommendations based on data to decision-makers (item #27), where at least two thirds of respondents across all group scored the item as high/very high (Tables 7, 8). One of the individual capacity factor items with the lowest level of agreement was staff having enough resources to evaluate data (item #22); more than half of respondents from government institutions scored it as very low/low. Other items related to resources (items #24, #26 and #28) were scored as low by government institutions, especially at the federal and state levels (Table 7). Development partners, however, generally scored items related to resources as high/very high. At the LGA level, regular access to the internet for data tasks (item #30) was the lowest-scoring item.

Levels of agreement on individual capacity factor items were generally consistent between the policy versus implementation agencies of the health sector. FMOH/SMOH respondents scored items related to resources for data-related tasks (items #22, #24, #26 and #28) lowest, while NPHCDA/SPHCDA respondents scored access to the internet lowest (item #30).

## KII results

Qualitative results are presented by the seven steps represented in the data demand and data use conceptual framework (Fig. 2; Table 1) [10]. As previously stated, the recognition and acquisition steps represent data demand. Cognition, discussion, reference, adaptation and influence reflect data use.

### Data demand: recognition

Recognition relates to the motivation and prioritization given to using data to answer questions with data. Across administrative levels, though less often at the LGA level, respondents appreciated data and saw their use as essential to address their different responsibilities in nutrition and other programs. The type of indicators or data sources used varied on the basis of the administrative level and type of decisions.

Data is a tool you cannot do without, not only in nutrition but in every aspect…You don’t know where we are going, so we need data to plan which age group? Which Community? What will you do with the resources? – Kaduna LGA respondent.

Some respondents did not see themselves as decision-makers. Rather, they saw high-level actors as the “decision-makers”. A few of these respondents, mainly at the federal level, thought high-level decision-makers or political leaders did not value using data in decision-making. This finding aligns with the quantitative findings, where a few federal- and LGA-level stakeholders disagreed with item #3 *(decision-makers in this institution give consideration to any recommendations based on data)* compared with none of the state and development partner respondents. The federal respondents explained that high-level actors sometimes made decisions based on personal assumptions because they did not believe data or dismissed them as unreliable due to quality issues.

When we present data to the decision makers and you give them an information from the survey, the very informed ones among them easily brush it aside – not to say that the situation doesn’t exist but maybe it’s been over estimated…because well, you guys could be wrong. – federal government respondent.

Other respondents attributed low levels of data appreciation among political leaders to the nutrition community’s own shortcomings in effectively using data for advocacy and helping leaders understand nutrition issues, an issue discussed further under the Adaptation step below.

### Data demand: acquisition

Acquisition reflects knowing where and how to access the data needed and having the resources to do so. Government and development partner respondents reported accessing nutrition data through published survey reports [for example, the Nigeria Demographics Health Survey (NDHS), Multiple Indicator Cluster Survey (MICS) and the National Health and Nutrition Survey], National Health Management Information System (NHMIS/DHIS-2) platform, facility registers or an FMOH data portal or from the FMOH’s Department of Planning, Research, and Statistics (DPRS). Many respondents indicated challenges with nutrition indicators being incomplete, of poor quality, or missing for routine administrative data. Development partners also relied on nutrition program data collected by their institutions or other development partners.

The reports for the national surveys were easily accessible, although some respondents reported long delays in the publishing of National Health and Nutrition Survey results. Respondents in all groups identified challenges accessing NHMIS/DHIS-2 administrative data on nutrition, in particular the time and effort required to access them. “You can’t access information when you want it, how you want it, without physically chasing people to get it, so all these things make it laborious and tiring”, explained one development partner respondent, for example. State and LGA government stakeholders discussed relying on M&E officers, nutrition focal points or other colleagues from within or outside their institutions to access administrative data. Some federal government and development partner respondents reported relying on friends or colleagues with NHMIS/DHIS-2 login credentials to obtain data on their behalf or asking state and LGA actors to gather information directly from facility registers.

Respondents from the FMOH-DPRS, who manage the NHMIS/DHIS-2, explained the need to limit platform access for security reasons. However, some federal government respondents who said they qualify for credentials per policy did not have them. Limited access to administrative data led some respondents to use survey data that they considered out of date.

When we were trying to write proposals for this large...funding program, and we didn’t have a presence in some of the states, it was impossible for us to obtain the routine data. So, everything was just quoting DHS data that was collected…in 2017. – development partner respondent.

Respondents identified a lack of resources, including insufficient human resources and materials such as data registers, as barriers to data acquisition. In Nigeria, facility workers provide health services and collect and report data. Insufficient human resources in facilities contribute to work overload and negatively impact facility-level data capture and reporting. Furthermore, registers for data capture were not produced and distributed in sufficient numbers. Respondents at all administrative levels, but especially LGA, reported a lack of electricity, computers and internet access in their workplace. They relied on personal computers and used personal finances to purchase internet access. These resource gaps impact both data entry and data retrieval from data management systems. This finding was also reflected in the quantitative results, where government stakeholders, especially those at LGA level, gave low scores to having access to computers and internet to support data work (items #29 and #30).

### Data use: cognition

Cognition is the ability to assess and understand available data and having the resources to support this capacity. Respondents discussed cognition in relation to analysing and presenting data and participating in trainings to do these tasks. Federal government respondents described quarterly or bi-annual trainings by DPRS on data extraction, analysis and presentation for state-level NHMIS/DHIS-2 focal persons. LGA-level respondents, including those in health facilities, said these trainings were infrequent and not all workers received them. They identified supportive supervision and quality assurance visits by federal and state representatives for on-the-job mentoring as the more common activity. A state-level respondent acknowledged that it was not possible, however, to reach all public and private facilities during these supervisory visits:

Even if you want to go to all of them in a month, I don’t know if that’s possible… If I have 35 public health facilities, I have over 300 private [facilities], even if I’m doing supervision well [for public facilities] but private facilities are doing it wrong, then I’m doing it wrong. – Kaduna state respondent.

Several facility-level respondents specifically discussed their capacity to assess data focusing on skills/tasks such as manual and electronic data collection, extraction of register data to NHMIS/DHIS-2 monthly summary forms, tracking of data on provided services and interpretation of charts.

### Data use: discussion

Discussion relates to opportunities to discuss and share data with others in different fora. State, LGA and development partner respondents talked more about participating in activities that promote the review and use of data with others, such as in quarterly data review meetings, compared with federal-level respondents. Survey findings also reflect this finding, as federal government respondents were less likely to highly agree that their institutions conduct activities to promote the use of data (item #5) compared with other groups.

State and LGA respondents described monthly meetings held to review, discuss and validate NHMIS/DHIS-2 data quality. These were carried out between facility and LGA-level staff or LGA and state-level staff. These monthly meetings fed into quarterly data review meetings where data are discussed with a wider group of stakeholders including different government ministries, divisions and agencies, development partners, civil society organizations, religious and traditional institutions.

We review and validate the data instead of waiting for quarterly review. So, by the time we come for quarterly meeting, our work would have been minimized… it will cumulate into better data quality for the state. – state-level respondent.

Although most respondents described the meetings as useful, some felt burdened by the number of required meetings.

Some federal government respondents discussed giving data quality feedback to lower administrative levels. A few federal respondents emphasized that data use is inherent in their institution’s work but not a specific activity:

From the way the organization is structured, it’s a given that we have to use data to because a lot of work we do have to do with advocacy, and we need data to really push whatever agenda it is that we are pushing. – federal government respondent.

### Data use: reference

“Reference” refers to the ability to interpret and synthesize data. Respondents across groups discussed interpreting different nutrition data to identify problems, set targets, assess progress, identify solutions or interventions and provide services. Data interpretation was generally seen as challenging. A few state-level respondents explained the need for collaboration between M&E officers, who possess data assessment skills, and nutrition program officers, who have technical expertise in nutrition, to facilitate data interpretation. A few respondents also identified a need to strengthen M&E officers’ overall data interpretation capacities. One state-level respondent explained:

It’s one thing to extract data correctly and it’s another thing to interpret that data, and to be able to take the appropriate decisions. Once the data is not properly interpreted you may take the wrong decision. – Kaduna state government respondent.

Poorly defined indicators, especially within the routine data system, low capacity for data users to triangulate multiple data points and a lack of investment in data interpretation were specific challenges related to reference voiced by respondents. Federal, state and development partner respondents noted that more resources were going into data production and less on the interpretation of existing information, especially at lower administrative levels. These respondents perceived that LGA and facilities reported NHMIS/DHIS-2 data to the state and federal levels without using it at their own level. They perceived this focus on transferring data from LGA to higher administrative levels as disincentivizing and devaluing data use at the LGA and facility levels. Simultaneously, LGA respondents, including health facility staff, expressed a desire to use data but reported limited capacity in nutrition generally and also in data analysis and interpretation. One LGA-level respondent explained:

I am not really fully trained on data analysis…at least if we are able to analyze our data, we will be able to make corrections and improvements [to] activities or services being rendered to the community. Because if you are able to analyze the data, at least we know what is going on. When we know what is going on, we know how to address the problem. – Lagos health facility respondent.

Some respondents at higher administrative levels noted that LGA and facility staff may interpret the absence of action or resource allocation as an indication the data they collect and share are not used. One LGA respondent described this dynamic:

Sometimes…you keep working and gathering the data and at the end of the day the data is not used. You feel your work is belittled. So, at the end of the day, even our field officers get discouraged because the data they pushed is not used to enhance the activities, so it causes discouragement. – Kaduna LGA respondent.

### Data use: adaptation

Adaptation is the ability to adapt information to local contexts. Themes related to adaptation did not come up often during KIIs, but as noted under recognition, some respondents suggested that poor data use and comprehension by high-level decision-makers was due to nutrition actors not appropriately contextualizing the data for them. A federal government respondent explained that data should communicate to decision-makers that “*this is the situation in your state or in your local government and in the country and this is what we need to do*” but that this was not often the case.

Still, one Lagos respondent explained that high-level decision-makers do not necessarily retain and apply data that are communicated to them and require more consistent reinforcement for collaborative decision-making:

Each office of decision-makers, there should be someone that resides within that office to re-emphasize and re-echo, “this is the decision we make; somebody came last time to tell you this is it, and we’re still there”. Each time program officers are invited to the meeting, [we] present data to the decision-makers and interpret…but once you’re done with that, that is where it ends. – Lagos state respondent.

### Data use: influence

Influence reflects whether there are incentives for decisions to be influenced by data. Respondents across groups expressed that data should influence decisions. They believed data should inform the work they do and be used as benchmarks for their goals. At the federal and state levels, data were used to inform development of policies and operation plans and to put issues in context (for example, which local government areas are affected by a certain issue or which topic is more of a pressing issue). Although LGA and facility respondents perceived the value of using data to guide decisions, they also sensed they lacked the space to use that information.

## Discussion

This mixed methods study provides information on the capacities to demand and use nutrition data among institutions working on nutrition within Nigeria’s health sector, as well as the facilitators and constraints faced in this capacity. The guiding conceptual framework for the capacity to demand and use data from Rodriguez et al. identifies different steps involved in the conceptualization of capacity to demand and use data [10].

The scale to assess the capacity to demand and use data showed institutional scores in the high 70s to mid-80s out of 100 across institutions at the federal, state, LGA and development partner levels. Development-partner- and state-level institutions scored items more highly than other groups, but the difference was not statistically significant. Similarly, primary healthcare institutions (policy implementers) scored items more highly than ministry institutions (policy formulators), but the difference was not significant. Government institutions lacked financial and material resources to sufficiently evaluate nutrition data and make recommendations based on data. Comparatively, development partner respondents reported challenges with having sufficient time to use data. These potential differences by organization type suggest that the nature of an organization is important to consider for the capacity to demand and use nutrition data in Nigeria. Previous studies have found that organizational type can influence access to, priorities for and interpretation of nutrition data [26].

In regard to the domains related to data demand, both quantitative and qualitative findings showed high recognition and support for using nutrition data to inform decision-making. Item-level analysis of the survey scales showed recognition of the role data can play within institutions. Respondents reported that the government and policy environment valued the use of data. The qualitative data, however, clarified that this high recognition was more in rhetoric than in practice. There was less transparency or understanding by respondents around how high-level actors used data in final decisions. These actors’ decision-making in nutrition was perceived by respondents to be less grounded in data. They were also perceived to not value or trust nutrition data, a finding that aligned with previous research that found a weak culture of evidence-based decision-making in Nigeria’s health sector [44]. Another study found that data are inadequately used in nutrition policy-making in Nigeria [45]. Respondents also highlighted the need for more robust advocacy on both nutrition as a topic and data use for high-level actors. They pointed out that there is often limited understanding of nutrition challenges and how to tackle these with available data. Better data interpretation and clearer evidence translation from the sector could better support effective actions.

KIIs also found interesting perceptions on who is seen as a “decision-maker”, especially among respondents from federal and local government institutions, who did not see themselves as decision-makers within their roles. Similar findings have been found in other LMICs, where health policy and program actors did not see themselves as decision-makers despite their roles in making decisions related to many aspects of program implementation [31, 46]. The results highlight the need to shift the narrative of nutrition data-informed decision-making away from just decisions made by high-level actors and to show how data are an integral part of a wider range of policy and program responsibilities at all levels of the system. A multi-country study found that nutrition data can effectively guide decision-making at national, subnational and local levels when deliberate efforts are made to develop a robust nutrition information system [47].

In terms of data acquisition, the second framework domain of data demand, both quantitative and qualitative results identified infrastructural and technological resource barriers that inhibited access to nutrition data. Consistent with findings from other LMICs, lack of access to computers, reliable internet networks and electricity in the work environment limited the collection, reporting, accessibility and therefore use of data [17, 18, 22, 29, 30, 48]. Technological interventions for the management of health information systems (for example, electronic data collection and reporting) have improved data quality and use at the facility level in Nigeria and other LMICs [22, 30, 49]. However, these interventions also pose feasibility challenges in low-resource settings that must be taken into consideration in their design and delivery [21]. Technology can be a great tool, but it requires continual technical support, maintenance and availability for its sustainability. Respondents in our study, especially those at health facilities and the LGA level, emphasized the importance of investing resources not just in data production but also in program implementation. Well-financed M&E or data teams without adequately resourced implementation teams will not improve population-level outcomes [23].

Accessing available nutrition data was a common challenge, especially for national surveys that were delayed in publishing reports, resulting in data not being available when needed. Respondents also described several data access challenges related to the routine administrative nutrition data in the NHMIS/DHIS-2 system. Respondents invested a lot of time and effort to access the platform only to find the information limited, incomplete or missing, which affects the trust and use of data [50]. Other studies assessing the availability, quality and use of data in health programs such as malaria, immunization and maternal, newborn and child health in Nigeria have consistently found challenges with the completeness and timeliness of available data, which negatively affects their subsequent use by decision-makers [22, 48, 51, 52]. Barriers such as the lack of reporting forms, limited human resources and staff overload, particularly at the facility level, also constrained data availability and access. Strengthening access to nutrition data in Nigeria is critical because, when decision-makers have access to quality and timely data, the data are more likely to be used in decision-making [13, 17, 18].

Both scale results and KIIs highlighted weak organizational mechanisms to ensure data use. In the survey scale, processes such as accountability mechanisms for data use or how institutional work aligns with what the data says, were less likely to be scored highly. Key informant interviews revealed that there were supervision visits and training on nutrition data collection, extraction, analysis and presentations; these were mainly top-down (federal-level actors to state or LGA actors) and ad hoc and did not cover many facilities or actors. The support services were also perceived to be more focused on data production rather than data use. About 10% of respondents across all groups of respondents reported their institutions lacked staff to interpret and synthesize data.

Subnational respondents were more likely to report engaging in discussions about nutrition data with others and through different fora, particularly through monthly and quarterly review meetings to assess NHMIS/DHIS-2 data. These review meetings informed strategic planning and service delivery in the two study states. These review meetings served as useful feedback mechanisms on the data collected and reported for LGA actors and health facilities in Kaduna and Lagos states.

Evidence shows that such review meeting activities can facilitate data demand and data use, specifically in nutrition and in the health sector in general, by encouraging shared motivation, shaping and reinforcing attitudes on data and developing stakeholders’ self-efficacy with data use [30, 50]. They facilitate dialogue, mutual learning and collaborative decision-making, which can strengthen organizational capacity and data culture [17, 30]. The systematic attention given to the data collected can also increase data quality and use over time [53]. Targeting technical support on nutrition data analysis, interpretation and synthesis during these review meetings could strengthen data demand and data use [54].

A key theme in the results was some respondents’ perceptions that the nutrition sector had a strong focus on data collection and production and less on data use, especially at the LGA levels. Respondents also highlighted challenges with interpreting, synthesizing and putting nutrition data into perspective to inform decisions. LGA-level respondents, for example, reported not using nutrition data optimally to inform local-level decisions due to low self-efficacy in both data analysis and nutrition services. There was more focus on ensuring data quality for upward reporting into the NHMIS/DHIS-2 administrative data system. This, however, did not translate into incentives for data interpretation or local-level decision-making. Studies in countries such as Malawi, Ghana and Tanzania have found that feedback loops and the use of routine administrative health data, especially at the facility level, was associated with improved data availability and completeness as facility actors valued this information more [19, 55].The lack of adequate data and nutrition skills was also evident at the state level. Nutrition program officers discussed the need to strengthen their data analysis and interpretation skills, while M&E officers lacked the nutrition expertise to synthesize information from nutrition data.

Weak organizational processes are a barrier to data use in decision-making [10, 39, 56]. However, well-articulated processes to access, assess, discuss, interpret and institutionalize data use in decision-making supports a culture for data demand and data use. Such processes can provide clear responsibility and accountability mechanisms and maintain capacities over time, despite turnover in personnel [8, 57, 58]. Training individuals in areas that promote data demand and data use is important, but it is not sufficient to affect change in data use [39, 56]. Therefore, advocacy through network building and resources for structures and processes that promote data use for nutrition-relevant decisions, such as the data review meetings conducted at the state and LGA levels, should be promoted [12, 14, 16].

Developing and investing resources in capacity at multiple levels, including at the facility level, is critical to strengthening Nigeria’s nutrition data value chain [2]. The results in this study highlight several areas that could inform capacity strengthening for institutions addressing nutrition and nutrition stakeholders, including development partners. Specifically, framing data use within the context of different types of decision-making processes can shine a light on the relevance of data use in different nutrition-relevant programmatic and policy decisions not just at a high political level. Investments in time, financial resources and technology (for example, computers and internet) are needed. Tools that improve nutrition data visibility to decision-makers, such as data visualization tools (DVT), could also be helpful to better identify the collected nutrition-relevant data and poorly defined indicators and to address gaps such as incomplete and missing data. Such tools have simplified the communication of data and increased data use, which influenced data quality and overall use for immunization in Nigeria [22]. Such tools are being developed for nutrition in Nigeria and their continual development and improvement could strengthen nutrition data visibility, use and quality [59–61]. Lastly, capacities on synthesizing and interpreting nutrition data should be strengthened to advance data use and data-informed decision-making across administrative levels. This will require institutions investing in and supporting the building of both nutrition and data skills.

Nutrition data come from a variety of sources such as multi-topic household surveys, ministry-specific management information systems and administrative data systems; these different sources contribute to the complex landscape of nutrition data [36]. Yet, there are limited studies on stakeholder perspectives on nutrition data access and use in LMICs [27]. Both government and development partner respondents in this study identified challenges in defining and harmonizing indicators across platforms, as well as in interpreting and translating information from disparate sources. Prioritizing key nutrition indicators to collect and assess regularly, as has been done in other health programs will be crucial improve data quality and usability [22, 62].

## Strengths and limitations

One strength of this study is that its mixed methods design integrates survey scale results with rich qualitative information. This study’s sampling strategy also provided information on the experiences and context of nutrition stakeholders across different administrative levels and in both government and non-government institutions in Nigeria. It allowed us to identify similarities and some important differences across administrative levels which can inform the design and targeting of interventions to strengthen capacities and promote the use of data in decision-making in nutrition. Another strength is that preliminary qualitative findings were shared with study participants, and their feedback helped to inform the final results.

Despite these strengths, this study also has important limitations. First, the sample size of the quantitative survey was small because we only focused on actors working on nutrition within the health and health-adjacent institutions. This focus on nutrition actors thereby limited the number of respondents eligible to respond to the study. Despite this limitation, our sample size was is similar to other studies looking at data use capacities in the health sector [10, 44, 52]. Second, at the subnational level, we sampled from two states with high political will to address nutrition and strong investments in nutrition and data. The experiences of these stakeholders in these two states may not reflect the typical experience in other states. Despite this, we believe that learning from the experience of these contexts provides useful information for future efforts to promote data demand and data use in decision-making. Another limitation is that social desirability could have contributed to how respondents, whether from government or development partner institutions, answer the survey scale given the low variability in responses to the organizational capacity factor. Combining the scale with qualitative interviews helped to address this limitation by enabling more in-depth discussions on the capacities to demand and use data in the nutrition sector.

## Conclusions

Better consideration and use of data can help address and better track lagging progress in addressing malnutrition in Nigeria and across LMICs [63]. However, research on the use of data for decision-making has not focused on nutrition. Rather, published studies in the global nutrition field generally reflect the use of research evidence for decision-making rather than data from population-level monitoring systems. There is a gap in understanding how routine, survey and surveillance nutrition data are collected, accessed, interpreted and used in the decision-making process.

Our study findings are consistent with other studies that have found that the demand and use of data in the health sector in Nigeria is limited, with data being used for some decisions but not others [41, 64]. There is broad recognition and support for the importance of data for nutrition decision-making across the different administrative levels of Nigeria’s health sector. Constraints remain, however, in individual capacity, resource availability and data use processes, particularly at the federal and LGA levels. Challenges such as limited access to quality and timely nutrition data and optimal nutrition indicators that are well-defined also continue. Inadequate training on nutrition and the analysis, synthesis and interpretation of nutrition need to be addressed. Insufficient infrastructure and weak feedback mechanisms hinder effective data use for the nutrition sector but also across the health sector. These challenges can weaken nutrition intervention design, proper targeting, sufficient coverage of nutrition and nutrition-relevant interventions, optimal management and the allocation of limited resources. Strengthening individual skills, improving the collection and access to data, ensuring consistent resources and fostering a culture of collaborative interpretation and use of data are essential to translating data into action for improved nutrition outcomes in Nigeria.

## Electronic Supplementary Material

Supplementary Material 1.

## Data Availability

The quantitative data described in the manuscript and analytic code will be made publicly and freely available without restriction at the DataDENT Dataverse. The qualitative data described in the manuscript will not be made available, because respondents participated with the understanding that interviews were confidential. The nature of the questions makes it that, even if names and organizations are removed from the transcripts, other information could act as identifiers. Lastly, ethical approval was also provided with information that stated that study investigators would not make public any information that could make it possible to identify interviewees. The codebook for qualitative can be made available upon request to the authors.
