# Supplementary material for: Assessing institutional capacities to demand and use nutrition data for decision-making in Nigeria’s health sector: A mixed-methods study
Source: Health Res Policy Syst. 2025 Sep 29;23:117. doi: 10.1186/s12961-025-01387-9 (PMC12482118; doi:10.1186/s12961-025-01387-9)
Supplement: Supplementary file 1 — Supplementary Material 1. [file 12961_2025_1387_MOESM1_ESM.docx]

**Supplementary 1: Full distribution of scores on organizational capacity items by administrative level & development partners**

|  |  | **Federal (n=33)** | | | | **State (n=21)** | | | | **LGA (n=29)** | | | | **Development Partner (n=9)** | | | | |
| --- | --- | --- | --- | --- | --- | --- | --- | --- | --- | --- | --- | --- | --- | --- | --- | --- | --- | --- |
| **Item** | **Level of agreement** | **LOW HIGH** | | | | **LOW HIGH** | | | | **LOW HIGH** | | | | **LOW HIGH** | | | |  |
|  |  | **0** | **1** | **2** | **3** | **0** | **1** | **2** | **3** | **0** | **1** | **2** | **3** | **0** | **1** | **2** | **3** |  |
| 1 | Using data is a priority in this institution. | 0.0% | 0.0% | 14.3% | 85.7% | 0.0% | 0.0% | 0.0% | 100.0% | 0.0% | 3.5% | 6.9% | 89.7% | 0.0% | 0.0% | 0.0% | 100.0% | |
| 2 | Leadership in this institution supports data-informed decisions. | 0.0% | 0.0% | 11.9% | 88.1% | 0.0% | 0.0% | 9.5% | 90.5% | 0.0% | 6.9% | 6.9% | 86.2% | 0.0% | 0.0% | 11.1% | 88.9% | |
| 3 | Decision-makers in this institution give consideration to any recommendations based on data. | 0.0% | 7.1% | 31.0% | 61.9% | 0.0% | 0.0% | 14.3% | 85.7% | 3.5% | 6.9% | 10.3% | 79.3% | 0.0% | 0.0% | 22.2% | 77.8% | |
| 4 | There is a transparent process for how data are used in decisions in this institution. | 0.0% | 4.8% | 42.9% | 52.4% | 0.0% | 4.8% | 19.1% | 76.2% | 0.0% | 10.3% | 17.2% | 72.4% | 0.0% | 0.0% | 33.3% | 66.7% | |
| 5 | This institution conducts activities to promote the use of data. | 0.0% | 7.1% | 23.8% | 69.0% | 0.0% | 0.0% | 19.1% | 81.0% | 0.0% | 0.0% | 17.2% | 82.8% | 0.0% | 0.0% | 11.1% | 88.9% | |
| 6 | This institution has a process to check regularly whether I use data in my work. | 7.1% | 28.6% | 33.3% | 31.0% | 0.0% | 14.3% | 38.1% | 47.6% | 0.0% | 6.9% | 13.8% | 79.3% | 0.0% | 33.3% | 33.3% | 33.3% | |
| 7 | This institution gets involved with researchers as partners in decision-making. | 0.0% | 7.1% | 28.6% | 64.3% | 0.0% | 14.3% | 28.6% | 57.1% | 3.5% | 13.8% | 48.3% | 34.5% | 0.0% | 11.1% | 22.2% | 66.7% | |
| 8 | This institution has a good process to advocate its priorities based on data to the public, such as to promote behavior change. | 0.0% | 4.8% | 40.5% | 52.4% | 0.0% | 4.8% | 19.1% | 76.2% | 3.5% | 3.5% | 20.7% | 72.4% | 0.0% | 0.0% | 33.3% | 66.7% | |
| 9 | This institution has a good process to advocate its priorities based on data to health workers, such as to promote changes in clinical practice. | 2.4% | 11.9% | 38.1% | 45.2% | 0.0% | 4.8% | 23.8% | 71.4% | 3.5% | 3.5% | 24.1% | 69.0% | 0.0% | 0.0% | 55.6% | 44.4% | |
| 10 | This institution has a good process to advocate its priorities based on data to other ministries or departments, such as to justify the costs of health interventions. | 0.0% | 9.5% | 19.0% | 71.4% | 0.0% | 5.0% | 30.0% | 65.0% | 3.5% | 3.5% | 31.0% | 62.1% | 0.0% | 11.1% | 0.0% | 88.9% | |
| 11 | This institution has a good process to advocate its priorities based on data to professional organizations, such as to promote new roles for different health workers. | 0.0% | 11.9% | 38.1% | 47.6% | 0.0% | 9.5% | 28.6% | 61.9% | 0.0% | 0.0% | 62.1% | 37.9% | 0.0% | 0.0% | 55.6% | 44.4% | |
| 12 | The current policy environment is supportive of this institution using data for its decisions. | 0.0% | 7.1% | 38.1% | 54.8% | 0.0% | 0.0% | 14.3% | 85.7% | 7.1% | 0.0% | 28.6% | 64.3% | 0.0% | 22.2% | 44.4% | 33.3% | |
| 13 | The current government is supportive of this institution using data for its decisions. | 0.0% | 7.1% | 45.2% | 47.6% | 0.0% | 0.0% | 14.3% | 85.7% | 0.0% | 0.0% | 17.2% | 82.8% | 0.0% | 11.1% | 55.6% | 33.3% | |
| 14 | Stakeholders outside this institution actively engage this institution to contribute data to inform decisions. | 0.0% | 7.1% | 35.7% | 57.1% | 0.0% | 9.5% | 28.6% | 61.9% | 0.0% | 10.3% | 27.6% | 62.1% | 0.0% | 22.2% | 33.3% | 44.4% | |
| 15 | Staff in this institution **search for and retriev**e data for the institution. | 2.4% | 7.1% | 26.2% | 64.3% | 0.0% | 0.0% | 38.1% | 61.9% | 6.9% | 0.0% | 27.6% | 65.5% | 0.0% | 0.0% | 0.0% | 100.0% | |
| 16 | Staff in this institution **interpret data** for this institution. | 2.4% | 7.1% | 40.5% | 50.0% | 0.0% | 0.0% | 28.6% | 71.4% | 0.0% | 3.5% | 24.1% | 72.4% | 0.0% | 0.0% | 22.2% | 77.8% | |
| 17 | Staff in this institution **synthesize all the relevant data**, information and analyses for a specific issue. | 0.0% | 11.9% | 31.0% | 57.1% | 0.0% | 4.8% | 38.1% | 57.1% | 3.5% | 6.9% | 24.1% | 65.5% | 0.0% | 11.1% | 22.2% | 66.7% | |
| 18 | Staff in this institution compare what this institution does to what the data say. | 0.0% | 11.9% | 35.7% | 52.4% | 0.0% | 9.5% | 19.1% | 71.4% | 0.0% | 10.3% | 17.2% | 72.4% | 0.0% | 0.0% | 33.3% | 66.7% | |
| 19 | Staff in this institution **link data to key issues** facing decision-makers. | 0.0% | 4.8% | 38.1% | 57.1% | 0.0% | 0.0% | 33.3% | 66.7% | 0.0% | 6.9% | 13.8% | 79.3% | 0.0% | 0.0% | 33.3% | 66.7% | |
| 20 | Staff in this institution provide recommendations based on data to decision-makers. | 0.0% | 2.4% | 26.2% | 71.4% | 0.0% | 0.0% | 19.1% | 81.0% | 0.0% | 3.5% | 41.4% | 55.2% | 0.0% | 0.0% | 33.3% | 66.7% | |

**Supplementary 2: Full distribution of scores on organizational capacity factor by ministry and primary care institutions**

|  |  | **Ministries of Health (n=25)** | | | | **Primary care agencies (n=46)** | | | |
| --- | --- | --- | --- | --- | --- | --- | --- | --- | --- |
| **Item** | **Level of agreement** | **LOW HIGH** | | | | **LOW HIGH** | | | |
|  |  | **0** | **1** | **2** | **3** | **0** | **1** | **2** | **3** |
| 1 | Using data is a priority in this institution. | 0.0% | 0.0% | 24.0% | 76.0% | 0.0% | 2.2% | 4.4% | 93.5% |
| 2 | Leadership in this institution supports data-informed decisions. | 0.0% | 0.0% | 20.0% | 80.0% | 0.0% | 4.4% | 4.4% | 91.3% |
| 3 | Decision-makers in this institution give consideration to any recommendations based on data. | 0.0% | 8.0% | 32.0% | 60.0% | 2.2% | 4.4% | 13.0% | 80.4% |
| 4 | There is a transparent process for how data are used in decisions in this institution. | 0.0% | 12.0% | 28.0% | 60.0% | 0.0% | 6.5% | 26.1% | 67.4% |
| 5 | This institution conducts activities to promote the use of data. | 0.0% | 8.0% | 40.0% | 52.0% | 0.0% | 0.0% | 13.0% | 87.0% |
| 6 | This institution has a process to check regularly whether I use data in my work. | 0.0% | 36.0% | 36.0% | 28.0% | 2.2% | 6.5% | 28.3% | 63.0% |
| 7 | This institution gets involved with researchers as partners in decision-making. | 0.0% | 12.0% | 40.0% | 48.0% | 2.2% | 10.9% | 34.8% | 52.2% |
| 8 | This institution has a good process to advocate its priorities based on data to the public, such as to promote behavior change. | 0.0% | 8.0% | 56.0% | 36.0% | 2.2% | 4.4% | 13.0% | 80.4% |
| 9 | This institution has a good process to advocate its priorities based on data to health workers, such as to promote changes in clinical practice. | 0.0% | 20.0% | 36.0% | 44.0% | 4.4% | 2.2% | 23.9% | 69.6% |
| 10 | This institution has a good process to advocate its priorities based on data to other ministries or departments, such as to justify the costs of health interventions. | 0.0% | 8.0% | 44.0% | 48.0% | 2.2% | 4.4% | 22.2% | 71.1% |
| 11 | This institution has a good process to advocate its priorities based on data to professional organizations, such as to promote new roles for different health workers. | 0.0% | 16.0% | 44.0% | 40.0% | 0.0% | 2.2% | 43.5% | 54.4% |
| 12 | The current policy environment is supportive of this institution using data for its decisions. | 0.0% | 0.0% | 28.0% | 72.0% | 4.4% | 0.0% | 24.4% | 71.1% |
| 13 | The current government is supportive of this institution using data for its decisions. | 0.0% | 4.0% | 36.0% | 60.0% | 0.0% | 0.0% | 17.4% | 82.6% |
| 14 | Stakeholders outside this institution actively engage this institution to contribute data to inform decisions. | 0.0% | 8.0% | 44.0% | 48.0% | 0.0% | 6.5% | 26.1% | 67.4% |
| 15 | Staff in this institution search for and retrieve data for the institution. | 0.0% | 4.0% | 64.0% | 32.0% | 4.4% | 2.2% | 19.6% | 73.9% |
| 16 | Staff in this institution interpret data for this institution. | 0.0% | 8.0% | 44.0% | 48.0% | 0.0% | 4.4% | 30.4% | 65.2% |
| 17 | Staff in this institution synthesize all the relevant data, information and analyses for a specific issue. | 0.0% | 12.0% | 44.0% | 44.0% | 2.2% | 6.5% | 28.3% | 63.0% |
| 18 | Staff in this institution compare what this institution does to what the data say. | 0.0% | 20.0% | 36.0% | 44.0% | 0.0% | 8.7% | 21.7% | 69.6% |
| 19 | Staff in this institution link data to key issues facing decision-makers. | 0.0% | 8.0% | 44.0% | 48.0% | 0.0% | 4.4% | 21.7% | 73.9% |
| 20 | Staff in this institution provide recommendations based on data to decision-makers. | 0.0% | 4.0% | 32.0% | 64.0% | 0.0% | 2.2% | 28.3% | 69.6% |

**Supplementary 3: Full distribution of scores on individual capacity factor by administrative level & development partners**

|  |  | **Federal (n=33)** | | | | **State (n=21)** | | | | **LGA (n=29)** | | | | **Development partner (n=9)** | | | | |
| --- | --- | --- | --- | --- | --- | --- | --- | --- | --- | --- | --- | --- | --- | --- | --- | --- | --- | --- |
| **Item** | **Level** **of agreement** | **LOW HIGH** | | | | **LOW HIGH** | | | | **LOW HIGH** | | | | **LOW HIGH** | | | |  |
|  |  | **0** | **1** | **2** | **3** | **0** | **1** | **2** | **3** | **0** | **1** | **2** | **3** | **0** | **1** | **2** | **3** |  |
| 21 | Our staff has enough time to evaluate data. | 6.1% | 24.2% | 48.5% | 21.2% | 4.8% | 9.5% | 57.1% | 28.6% | 17.2% | 31.0% | 27.6% | 24.1% | 0.0% | 44.4% | 33.3% | 22.2% | |
| 22 | Our staff has enough resources to evaluate data. | 33.3% | 42.4% | 18.2% | 6.1% | 9.5% | 57.1% | 14.3% | 19.1% | 20.7% | 31.0% | 31.0% | 17.2% | 0.0% | 11.1% | 44.4% | 44.4% | |
| 23 | Our staff has enough time to compare what this institution does to what the data say. | 6.1% | 30.3% | 42.4% | 21.2% | 0.0% | 28.6% | 33.3% | 38.1% | 17.2% | 20.7% | 37.9% | 24.1% | 0.0% | 44.4% | 22.2% | 33.3% | |
| 24 | Our staff has enough resources to compare what this institution does to what the data say. | 30.3% | 36.4% | 27.3% | 6.1% | 9.5% | 52.4% | 19.1% | 19.1% | 13.8% | 34.5% | 37.9% | 13.8% | 11.1% | 11.1% | 33.3% | 44.4% | |
| 25 | Our staff has enough time to link data to key issues facing decision-makers. | 3.0% | 30.3% | 39.4% | 27.3% | 0.0% | 33.3% | 33.3% | 33.3% | 6.9% | 27.6% | 41.4% | 24.1% | 0.0% | 11.1% | 77.8% | 11.1% | |
| 26 | Our staff has enough resources to link data to key issues facing decision-makers. | 27.3% | 36.4% | 27.3% | 9.1% | 4.8% | 38.1% | 33.3% | 23.8% | 6.9% | 34.5% | 37.9% | 20.7% | 0.0% | 0.0% | 55.6% | 44.4% | |
| 27 | Our staff has enough time to provide recommendations based on data to decision-makers. | 3.0% | 24.2% | 39.4% | 33.3% | 4.8% | 9.5% | 47.6% | 38.1% | 3.5% | 27.6% | 48.3% | 20.7% | 0.0% | 0.0% | 55.6% | 44.4% | |
| 28 | Our staff has enough resources to provide recommendations based on data to decision-makers. | 24.2% | 36.4% | 33.3% | 6.1% | 9.5% | 38.1% | 33.3% | 19.1% | 13.8% | 37.9% | 27.6% | 20.7% | 0.0% | 0.0% | 33.3% | 66.7% | |
| 29 | Our unit has regular access to a computer for acquiring and analyzing data. | 15.2% | 30.3% | 21.2% | 33.3% | 4.8% | 23.8% | 14.3% | 57.1% | 37.9% | 13.8% | 13.8% | 34.5% | 0.0% | 0.0% | 0.0% | 100.0% | |
| 30 | Our unit has regular access to the Internet at work for accessing data online. | 3.0% | 33.3% | 30.3% | 33.3% | 23.8% | 14.3% | 23.8% | 38.1% | 41.4% | 24.1% | 10.3% | 24.1% | 0.0% | 0.0% | 0.0% | 100.0% | |

**Supplemental 4: Full distribution of scores on individual capacity factor by ministry and primary care institutions**

|  |  | **Ministries of Health (n=25)** | | | | **Primary care agencies (n=46)** | | | | |
| --- | --- | --- | --- | --- | --- | --- | --- | --- | --- | --- |
| **Item** | **Level of agreement** | **LOW HIGH** | | | | **LOW HIGH** | | | |  |
|  |  | **0** | **1** | **2** | **3** | **0** | **1** | **2** | **3** |  |
| 21 | Our staff has enough time to evaluate data. | 8.0% | 20.0% | 60.0% | 12.0% | 13.0% | 23.9% | 37.0% | 26.1% | |
| 22 | Our staff has enough resources to evaluate data. | 40.0% | 48.0% | 8.0% | 4.0% | 15.2% | 39.1% | 30.4% | 15.2% | |
| 23 | Our staff has enough time to compare what this institution does to what the data say. | 8.0% | 36.0% | 36.0% | 20.0% | 10.9% | 23.9% | 32.6% | 32.6% | |
| 24 | Our staff has enough resources to compare what this institution does to what the data say. | 32.0% | 44.0% | 20.0% | 4.0% | 10.9% | 39.1% | 34.8% | 15.2% | |
| 25 | Our staff has enough time to link data to key issues facing decision-makers. | 4.0% | 40.0% | 36.0% | 20.0% | 4.4% | 26.1% | 37.0% | 32.6% | |
| 26 | Our staff has enough resources to link data to key issues facing decision-makers. | 28.0% | 48.0% | 16.0% | 8.0% | 4.4% | 34.8% | 41.3% | 19.6% | |
| 27 | Our staff has enough time to provide recommendations based on data to decision-makers. | 4.0% | 28.0% | 44.0% | 24.0% | 4.4% | 19.6% | 41.3% | 34.8% | |
| 28 | Our staff has enough resources to provide recommendations based on data to decision-makers. | 28.0% | 44.0% | 20.0% | 8.0% | 8.7% | 37.0% | 34.8% | 19.6% | |
| 29 | Our unit has regular access to a computer for acquiring and analyzing data. | 16.0% | 36.0% | 20.0% | 28.0% | 23.9% | 19.6% | 15.2% | 41.3% | |
| 30 | Our unit has regular access to the Internet at work for accessing data online. | 12.0% | 20.0% | 32.0% | 36.0% | 32.6% | 26.1% | 13.0% | 28.3% | |
